# Barriers to human papillomavirus vaccine uptake: role of state religiosity and healthcare professionals’ participation in a state vaccine program

**DOI:** 10.1093/jncics/pkad068

**Published:** 2023-09-12

**Authors:** Monalisa Chandra, Ikponmwosa Osaghae, Rajesh Talluri, Sanjay Shete

**Affiliations:** Department of Epidemiology, The University of Texas MD Anderson Cancer Center, Houston, TX, USA; Department of Epidemiology, The University of Texas MD Anderson Cancer Center, Houston, TX, USA; Department of Biostatistics, The University of Texas MD Anderson Cancer Center, Houston, TX, USA; Department of Data Science, University of Mississippi Medical Center, Jackson, MS, USA; Department of Epidemiology, The University of Texas MD Anderson Cancer Center, Houston, TX, USA; Department of Biostatistics, The University of Texas MD Anderson Cancer Center, Houston, TX, USA; Division of Cancer Prevention and Population Sciences, The University of Texas MD Anderson Cancer Center, Houston, TX, USA

## Abstract

**Background:**

Despite the known benefits of preventing human papillomavirus (HPV)–related cancers, HPV vaccine coverage is low in the United States. Grounded in Social Ecological theory, we assessed the macro-level (state) and meso-level (organization) factors associated with HPV vaccine initiation and up-to-date.

**Methods:**

Data from 2020 National Immunization Survey-Teen were used to study a sample of 20 163 US adolescents (aged 13-17 years). The data were collected from each teen’s parents or guardians and health-care professionals. Weighted prevalence estimates were calculated, and multivariable regression analyses were conducted.

**Results:**

The prevalence of HPV vaccine initiation was 75.1% and of remaining up-to-date was 58.6%. At the macro level, teens living in states with high and moderate religiosity had lower odds of HPV vaccine initiation (high religiosity adjusted odds ratio [AOR] = 0.63, 95% confidence interval [CI] = 0.50 to 0.78; moderate religiosity AOR = 0.68, 95% CI = 0.55 to 0.85) and up-to-date (high religiosity AOR = 0.69, 95% CI = 0.56 to 0.85; moderate religiosity AOR = 0.74, 95% CI = 0.61 to 0.91) than states with low religiosity. At the meso level, when none of their healthcare professionals ordered vaccine from the state, teens had lower odds of initiation (AOR = 0.68, 95% CI = 0.53 to 0.87) and up-to-date (AOR = 0.76, 95% CI = 0.60 to 0.95) than teens whose healthcare professionals ordered vaccine from the state. In addition, race and ethnicity, age, mother’s education level, household income, well-child examination status, and doctor’s recommendation were significantly associated with HPV vaccine uptake.

**Conclusion:**

A multiprong approach is needed to address religious and systemic barriers to HPV vaccination and expand healthcare professionals’ access and enrollment in state vaccine initiatives, such as the Vaccine for Children program.

Human papillomavirus (HPV) is responsible for common infections such as genital warts; papillomatosis; and multiple cancers, including oropharyngeal and anogenital cancers (1). Yearly, HPV accounts for almost 50000 new HPV-related cancer cases in the United States (2). The oropharynx is currently the most common site of HPV-related cancers in the United States (3), but cervical cancer is the most common HPV-related cancer among women, and oropharyngeal cancer is the most common HPV-related cancer among men (2). Cancers caused by HPV are among the few vaccine-preventable cancers (4). A safe and effective HPV vaccine has been available in the United States since 2006 (5), and it can prevent 90% of HPV-related cancers and genital precancers (6). Therefore, as a preventative measure, HPV vaccination is routinely recommended for all children 11-12 years of age, although the vaccination series can start at 9 years of age (7).

Despite the benefits, HPV vaccination coverage is suboptimal in the United States (8). Although Healthy People 2030 recommends that 80% of age-eligible adolescents receive the recommended dose of vaccine, only 75.1% of US adolescents had initiated (had at least 1 dose), and 58.6% were up-to-date on the vaccination series as of 2020 (ie, completed the recommended number of doses) (9,10). Multiple individual-level factors, such as clinician recommendation of vaccine, sex of the teen, well-child exam status, mother’s education level, household income, multiple clinician contacts in the past year, insurance coverage, race and ethnicity, vaccine safety, HPV and vaccine knowledge, and parents’ perceived severity account for low initiation and being up-to-date in the United States (11-13). In addition, individual religious beliefs and practices tend to influence vaccine receipt because of the faith-based objection to specific contents in the vaccines (eg, fetal cells used in SARS-CoV-2 vaccines), greater faith in religious means of healing over modern medicine, and lack of awareness of efficacy and effectiveness of the vaccine (14-18).

Vaccination initiation and being up-to-date do not depend on individual factors alone. The Social Ecological theory posits that individuals and the social environment are interconnected, and health behavior results from an interplay of factors at the individual level and the social environment (which includes interpersonal, organizational, community, and public policy factors) (19). HPV vaccination behavior has primarily been studied in the context of individual characteristics of teens, parents, or healthcare professionals (11,20-25). Macro-level characteristics, however, such as state religious ideology, have shaped health policies in the United States. Forty-four US states and Washington, DC, currently allow a religious exemption for mandatory vaccines (26). Moreover, in conservative religious groups, beliefs regarding appropriate sexual behaviors and taboos on sex were associated with hesitancy in HPV vaccine use (27). However, the role of the state’s religious ideology on HPV vaccine initiation and being up-to-date is understudied.

In addition to the macro-level factors, health behavior is affected by meso-level factors, such as the vaccine procurement process in the practicing organizations and facility type. To address the cost-related barriers to vaccination coverage, the Vaccines for Children (VFC) program was created (28). Studies have shown stronger recommendations for HPV vaccine by the healthcare professionals enrolled in the program (29), but there is a dearth of information about how the VFC program has influenced HPV vaccine coverage among adolescents in the United States. Although information about the effect of facility type on HPV vaccination is limited, clinics (for sexually transmitted diseases, school, and teen) or other facilities, and mixed facilities were identified as important determinants of HPV vaccination for male adolescents (30).

Because HPV vaccination and initiation studies have focused primarily on individual factors, there is a paucity of information about the role of macrostructural (state religiosity) and mesostructural (VFC enrollment/facility type) factors on vaccination coverage. Therefore, this study was grounded in Social Ecological theory to comprehensively understand the interplay of barriers and facilitators with respect to HPV vaccination, focusing on religiosity at the state level and source of vaccine procurement and facility type at the organizational level. We hypothesized that after adjusting for other individual-level factors, 1) highly religious states have low odds of HPV vaccination initiation and being up-to-date, 2) patients visiting all nonpublic facilities have higher odds of initiation of and being up-to-date with HPV vaccination, and 3) teens whose healthcare professionals procure vaccine from the state (ie, are enrolled in the VFC program) have higher odds of initiation of and being up-to-date with HPV vaccination.

## Methods

### Study population and data collection

The current study is based on a comprehensive dataset that included individual and state-level data. The individual-level data for the study were obtained from the 2020 National Immunization Survey-Teen (NIS-Teen 2020), a population-based survey that collected data on teenage vaccination from parents and guardians as well as healthcare professionals in all 50 states, the District of Columbia, and some US territories. The parents were interviewed regarding their teen's vaccination history and families’ sociodemographic factors by telephone. Then, each teen’s healthcare professional was contacted (with parental consent) to collect data on the immunization history using a mailed questionnaire. The details of the NIS-Teen 2020 study methodology were reported in previous publications (31). The National Center for Health Statistics research ethics review board approved the NIS-Teen 2020.

### Measures

#### Study outcome

The current study had 2 primary outcomes—HPV vaccination initiation and being up-to-date—that were obtained from clinician-verified vaccination history. Initiation of HPV vaccination was defined as receiving at least 1 dose of the HPV vaccine. The second outcome variable, being up-to-date, was defined according to the 2016 guidelines of the Advisory Committee on Immunization Practices based on 2 criteria: 1) receipt of 3 or more doses or 2) receipt of 2 doses of the HPV vaccine when the first shot was administered before the patient was 15 years of age (7).

#### Primary predictors

Information about the macro-level predictor, state religiosity, was obtained from the Religious Landscape Study by the PEW Research Center (32), developed by combining 4 individual measures of religious observance: self-assessment of religion’s importance in one’s life, religious attendance, frequency of prayer, and belief in God. The responses (‒1 = low, 0 = medium, and 1 = high) were then added to create the religiosity scale of the individual, where a score of +2 or higher was highly religious, a score between ‒1 and +1 was moderately religious, and a score of ‒2 was low religiosity. The details of the scale have previously been published (32). Based on the percentage of highly religious people, each state was then categorized as low religiosity (ie, presence of highly religious people ≤49%), moderate religiosity (ie, presence of highly religious people between 50% and 59%), and high religiosity (ie, presence of highly religious people ≥60%).

Meso-level predictors included 1) the type of facility in which the health-care professional practiced, which was assessed using the question “Which of the following describes this facility” (available responses were “All public facilities,” “All hospital facilities,” “All private facilities,” “All sexually transmitted disease/school/teen clinics or other facilities,” and “Mixed”) and 2) physicians ordering vaccine from the state. The second variable was used as a proxy to measure participation of healthcare professionals in the VFC program, assessed based on responses to the question “Do the teen’s providers order vaccines from state/local health department?” Given that some teens have multiple health-care professionals, this variable was categorized as “All providers,” “Some but possibly or definitely not all providers,” or “No providers” order vaccine from the state or local health department.

#### Individual characteristics

The following individual characteristics of teens were also assessed as predictor variables:

- **Receipt of a well-child exam.** This characteristic was measured using the question “Did teen have an 11-12-year-old well child exam or check-up?” Responses included in the study were “yes” or “no.”
- **Recommendation of HPV vaccine by a health-care professional.** This characteristic was measured using the question “Has a doctor or other health care professional ever recommended that teen receive HPV shots?” Responses included “yes,” “no,” and “don’t know.”
- **Respondents’ relationship to teen.** This characteristic was measured using the question, “What is your relationship to the teen?” Responses included “Mother or female guardian,” “Father or male guardian,” “Grandparent,” and “Other family members/friend.”

Additional individual characteristics included in the analysis were the teen’s age (continuous variable), sex (binary variable), race and ethnicity (categorical variable), family’s poverty status (categorical variable), and mother’s education level (categorical variable). Race and ethnicity were combined as a single variable based on the current literature (11,13,33,34).

### Statistical analysis

All analyses were survey weighted to account for the complex survey design in the NIS-Teen 2020 and allow for the representativeness of the demographics of the US population. Survey weights accounted for nonresponse, nonresolution of telephone numbers, subsampling of 1 age-eligible child per household, and multiple telephone lines in the home. Moreover, adjustments were made after stratification on the basis of the respondents’ sociodemographic characteristics and missing clinician data. The weighted prevalence of initiation of and being up-to-date with HPV vaccination were estimated. A survey-weighted multivariable logistic regression was conducted to determine the key factors associated with the initiation of and being up-to-date with HPV vaccination. The variables included in our model were selected a priori on the basis of previous literature and the study objective. We did not conduct any model selection. Because missingness for the variables included in our study was less than 2%, we conducted a complete case analysis. We also mapped the relationship between state religiosity and HPV vaccine initiation and up-to-date status across the US states. We set statistical significance as a 2-sided *P* < .05 for all tests included in this study. All statistical analyses were performed using R, version 4.2.3 (R Foundation for Statistical Computing, Vienna, Austria).

## Results

Table 1 shows weighted percentages and associated confidence intervals (CIs) of teens in the overall population and stratified by HPV vaccination initiation and up-to-date status on the basis of the NIS-Teen 2020 data. A total of 20 163 teens 13-17 years of age were included in the study. The average (SD) age of the teens in the study was 15 (1.4) years, 51% were male, 50% were non-Hispanic White, 19.6% had a family income below the federal poverty level, and 12.4% of teens’ mothers had less than 12 years of education. About 74.8% of teens received HPV vaccination recommendations from their physician, 85.3% of the teens had had an 11- to 12-year-old well-child exam or checkup, and 66.5% of the survey respondents were mothers or female guardians. About 55% of teens’ health-care professionals practiced in private facilities. About three-fourths (74.5%) of teens had all clinicians ordering vaccine from the state or local health department. About 34% of the teens came from highly religious states, 33.7% belonged to moderately religious states, and about 32% were from states with low religiosity.

**Table 1.** Characteristics of the participants in the overall population, stratified by initiation of and being up-to-date with HPV vaccination and based on 2020 National Immunization Survey-Teen (N = 20 163)^a^

Characteristic			% Weighted (95% CI)			
	Total		Initiation		Up-to-date	
	No. (weighted No.)	% Weighted (95% CI)	Yes	No	Yes	No
**Teen’s HPV vaccination**						
Initiation						
Yes	15 169 (15 697 001)	75.1 (73.9 to 76.2)				
No	4994 (5 211 255)	24.9 (23.8 to 26.1)				
Up-to-date						
Yes	12 115 (12 258 618)	58.6 (57.3 to 60.0)				
No	8048 (8 649 638)	41.4 (40.0 to 42.7)				
**Individual characteristics of teens**						
Age, mean (SD), y	20 163 (20 908 256)	15.0 (1.40)	15.0 (1.4)	14.8 (1.41)	15.1 (1.36)	14.8 (1.43)
Sex						
Male	10 587 (10 671 879)	51.0 (49.7 to 52.4)	73.1 (71.5 to 74.8)	26.9 (25.2 to 28.5)	56.0 (54.1 to 57.8)	44.0 (42.2 to 45.9)
Female	9576 (10 236 377)	49.0 (47.6 to 50.3)	77.1 (75.4 to 78.7)	22.9 (21.3 to 24.6)	61.4 (59.5 to 63.3)	38.6 (36.7 to 40.5)
Ethnicity and race						
Non-Hispanic White	12 585 (10 461 520)	50.0 (48.7 to 51.3)	71.1 (69.7 to 72.5)	28.9 (27.5 to 30.3)	55.4 (53.9 to 56.9)	44.6 (43.1 to 46.1)
Non-Hispanic Black	1667 (2 745 973)	13.1 (12.2 to 14.1)	78.1 (74.7 to 81.2)	21.9 (18.8 to 25.3)	60.7 (56.9 to 64.4)	39.3 (35.6 to 43.1)
Hispanic	3410 (5 233 713)	25.0 (23.7 to 26.4)	80.0 (77.0 to 82.7)	20.0 (17.3 to 23.0)	62.7 (59.3 to 66.1)	37.3 (33.9 to 40.7)
Multiple/others	2501 (2 467 050)	11.8 (11.0 to 12.7)	78.0 (74.6 to 81.0)	22.0 (19.0 to 25.4)	61.3 (57.5 to 64.9)	38.7 (35.1 to 42.5)
Family’s federal poverty status						
Below federal poverty level	2848 (3 845 030)	19.6 (18.4 to 20.9)	83.2 (80.8 to 85.4)	16.8 (14.6 to 19.2)	63.1 (59.7 to 66.4)	36.9 (33.6 to 40.3)
Above federal poverty level and income ≤$75 000	5567 (6 212 890)	31.7 (30.4 to 33.0)	72.5 (70.2 to 74.7)	27.5 (25.3 to 29.8)	54.1 (51.5 to 56.5)	45.9 (43.5 to 48.5)
Above federal poverty level and income >$75 000	11 037 (9 533 029)	48.7 (47.3 to 50.0)	74.3 (72.7 to 75.7)	25.7 (24.3 to 27.3)	60.2 (58.5 to 61.9)	39.8 (38.1 to 41.5)
Mother’s education level						
<12 y	1689 (2 590 351)	12.4 (11.3 to 13.6)	84.3 (80.8 to 87.3)	15.7 (12.7 to 19.2)	64.1 (59.2 to 68.7)	35.9 (31.3 to 40.8)
12 y	3058 (4 447 142)	21.3 (20.1 to 22.5)	72.6 (69.5 to 75.5)	27.4 (24.5 to 30.5)	55.0 (51.8 to 58.2)	45.0 (41.8 to 48.2)
>12 y, noncollege graduate	5187 (5 028 230)	24.0 (22.9 to 25.2)	71.2 (68.7 to 73.5)	28.8 (26.5 to 31.3)	54.1 (51.5 to 56.7)	45.9 (43.3 to 48.5)
College graduate	10 229 (8 842 533)	42.3 (41.0 to 43.6)	75.9 (74.3 to 77.3)	24.1 (22.7 to 25.7)	61.4 (59.7 to 63.1)	38.6 (36.9 to 40.3)
Doctor’s recommendation						
Has a doctor or other health-care professional ever recommended that teen receive HPV shots?						
Yes	15 419 (15 261 169)	74.8 (73.5 to 76.0)	80.8 (79.6 to 81.9)	19.2 (18.1 to 20.4)	65.3 (63.8 to 66.7)	34.7 (33.3 to 36.2)
No	3115 (3 484 851)	17.1 (16.0 to 18.2)	51.5 (48.0 to 54.9)	48.5 (45.1 to 52.0)	33.5 (30.3 to 36.9)	66.5 (63.1 to 69.7)
Don’t know	1263 (1 665 968)	8.2 (7.3 to 9.1)	74.9 (69.9 to 79.3)	25.1 (20.7 to 30.1)	54.8 (49.0 to 60.4)	45.2 (39.6 to 51.0)
Well-child examination for 11- and 12-year-olds						
Did teen have an 11- to 12-year-old well-child exam or check-up?						
Yes	10 025 (10 212 571)	85.3 (84.0 to 86.6)	80.8 (79.3 to 82.1)	19.2 (17.9 to 20.7)	65.1 (63.2 to 66.8)	34.9 (33.2 to 36.8)
No	1696 (1 754 680)	14.7 (13.4 to 16.0)	64.1 (59.6 to 68.3)	35.9 (31.7 to 40.4)	42.0 (37.4 to 46.8)	58.0 (53.2 to 62.6)
Relationship of the respondent to the teen						
Mother or female guardian	14 573 (13 901 875)	66.5 (65.2 to 67.8)	75.3 (73.9 to 76.7)	24.7 (23.3 to 26.1)	58.6 (57.0 to 60.1)	41.4 (39.9 to 43.0)
Father or male guardian	4777 (5 647 369)	27.0 (25.8 to 28.3)	72.9 (70.3 to 75.3)	27.1 (24.7 to 29.7)	57.4 (54.6 to 60.1)	42.6 (39.9 to 45.4)
Grandparent	469 (794 837)	3.8 (3.3 to 4.4)	83.6 (78.1 to 87.9)	16.4 (12.1 to 21.9)	67.2 (60.1 to 73.5)	32.8 (26.5 to 39.9)
Other family members/friends	341 (557 877)	2.7 (2.2 to 3.2)	79.4 (72.0 to 85.3)	20.6 (14.7 to 28.0)	60.8 (52.2 to 68.9)	39.2 (31.1 to 47.8)
**Meso-level characteristics**						
Facility type						
Which of the following describes this facility?						
All public facilities	2460 (2 491 936)	13.8 (12.8 to 14.8)	74.1 (70.5 to 77.3)	25.9 (22.7 to 29.5)	51.7 (47.6 to 55.7)	48.3 (44.3 to 52.4)
All hospital facilities	2756 (2 223 957)	12.3 (11.5 to 13.1)	76.0 (72.9 to 78.8)	24.0 (21.2 to 27.1)	61.2 (57.8 to 64.5)	38.8 (35.5 to 42.2)
All private facilities	9027 (9 909 221)	54.8 (53.4 to 56.1)	76.8 (75.2 to 78.3)	23.2 (21.7 to 24.8)	61.4 (59.5 to 63.2)	38.6 (36.8 to 40.5)
All sexually transmitted disease/school/teen clinics or other facilities	505 (473 979)	2.6 (2.2 to 3.1)	55.5 (47.4 to 63.4)	44.5 (36.6 to 52.6)	42.6 (35.0 to 50.5)	57.4 (49.5 to 65.0)
Mixed type	3356 (2 992 116)	16.5 (15.6 to 17.5)	75.9 (72.8 to 78.6)	24.1 (21.4 to 27.2)	59.1 (55.9 to 62.2)	40.9 (37.8 to 44.1)
Doctors order vaccine from state or local health department						
Does your practice order vaccine from your state or local health department to administer to children?						
All healthcare professionals	13 353 (12 943 350)	74.5 (73.2 to 75.7)	77.5 (76.1 to 78.7)	22.5 (21.3 to 23.9)	60.9 (59.4 to 62.5)	39.1 (37.5 to 40.6)
Some but possibly or definitely not all healthcare professionals	2185 (2 171 514)	12.5 (11.6 to 13.4)	77.4 (73.7 to 80.7)	22.6 (19.3 to 26.3)	57.2 (53.2 to 61.1)	42.8 (38.9 to 46.8)
No healthcare professionals	1975 (2 268 783)	13.1 (12.1 to 14.1)	68.3 (64.4 to 72.0)	31.7 (28.0 to 35.6)	52.1 (48.0 to 56.2)	47.9 (43.8 to 52.0)
**Macro-level characteristics**						
State religious ideology						
High religiosity	5956 (7 124 100)	34.1 (33.3 to 35.0)	72.0 (70.2 to 73.8)	28.0 (26.2 to 29.8)	53.5 (51.4 to 55.6)	46.5 (44.4 to 48.6)
Moderate religiosity	7570 (7 042 954)	33.7 (32.9 to 34.5)	74.3 (72.5 to 76.1)	25.7 (23.9 to 27.5)	58.3 (56.3 to 60.3)	41.7 (39.7 to 43.7)
Low religiosity	6233 (6 715 523)	32.2 (31.2 to 33.2)	79.0 (76.5 to 81.3)	21.0 (18.7 to 23.5)	64.4 (61.5 to 67.1)	35.6 (32.9 to 38.5)

^a^CI = confidence interval; HPV = human papillomavirus.

### Initiation

The overall initiation rate for the HPV vaccination series among adolescents was 75.1% (Table 1). The initiation prevalence among teens was highest for states with low religiosity (79%) compared with states with moderate (74.3%) or high religiosity (72%) (Table 1; Supplementary Figure 1, available online). Prevalence of initiation was highest when all health-care professionals ordered vaccine from the state or local health department (77.5%).

In the multivariable regression analyses (Table 2), at the macro level, we found that religiosity was a significant factor in HPV vaccine initiation. Compared with the teens living in states with low religiosity, those living in states with high religiosity and moderate religiosity had 37% lower odds (adjusted odds ratio [AOR] = 0.63, 95% CI = 0.50 to 0.78, *P* < .001) and 32% lower odds (AOR = 0.68, 95% CI = 0.55 to 0.85, *P* = .001) of HPV vaccine initiation, respectively.

**Table 2.** Multivariable survey-weighted logistic regression analyses correlating factors associated with initiation of and being up-to-date with HPV vaccination series among teens, based on 2020 National Immunization Survey-Teen^a^

Characteristic	Initiation				Up-to-date			
	AOR	Lower 95% CI	Upper 95% CI	*P*	AOR	Lower 95% CI	Upper 95% CI	*P*
**Individual-level characteristics, teens**								
Age	1.15	1.08	1.23	<.001	1.28	1.21	1.35	<.001
Sex								
Female	1.08	0.91	1.28	.363	1.14	0.98	1.33	.099
Male	(Referent)	N/A	N/A	N/A	(Referent)	N/A	N/A	N/A
Ethnicity and race								
Hispanic	1.84	1.42	2.38	<.001	1.52	1.21	1.90	<.001
Non-Hispanic Black	1.89	1.32	2.69	<.001	1.36	1.04	1.77	.027
Multiple/others	1.61	1.21	2.13	.001	1.36	1.04	1.77	.023
Non-Hispanic White	(Referent)	N/A	N/A	N/A	(Referent)	N/A	N/A	N/A
Family’s federal poverty level status								
Below federal poverty level	1.95	1.42	2.67	<.001	1.76	1.33	2.32	<.001
Above federal poverty level and income ≤$75 000	1.06	0.85	1.33	.607	0.98	0.80	1.20	.843
Above federal poverty level and income >$75 000	(Referent)	N/A	N/A	N/A	(Referent)	N/A	N/A	N/A
Mother’s education								
12 y	0.44	0.30	0.66	<.001	0.78	0.52	1.16	.213
>12 y, noncollege graduate	0.45	0.30	0.68	<.001	0.67	0.45	1.01	.055
College graduate	0.59	0.39	0.90	.013	1.01	0.67	1.51	.983
<12 y	(Referent)	N/A	N/A	N/A	(Referent)	N/A	N/A	N/A
Respondent’s relation to the teen								
Father or male guardian	1.20	0.98	1.47	.081	1.06	0.88	1.28	.529
Grandparents	1.55	0.84	2.87	.159	1.39	0.86	2.25	.18
Other family member/friends	2.09	1.07	4.09	.031	1.70	0.97	2.98	.065
Mother	(Referent)	N/A	N/A	N/A	(Referent)	N/A	N/A	N/A
Doctor recommended HPV vaccine								
Has a doctor or other health-care professional ever recommended that teen receive HPV shots?								
Yes	5.54	4.46	6.88	<.001	3.92	3.17	4.85	<.001
Don’t know	2.74	1.89	4.00	<.001	2.11	1.42	3.14	<.001
No	(Referent)	N/A	N/A	N/A	(Referent)	N/A	N/A	N/A
Well-child examination for 11- and 12-year-olds								
Yes	2.32	1.83	2.95	<.001	2.66	2.10	3.37	<.001
No	(Referent)	N/A	N/A	N/A	(Referent)	N/A	N/A	N/A
**Meso-level characteristic**								
Facility type								
All hospital facilities	0.93	0.65	1.35	.718	1.31	0.92	1.89	.138
All private facilities	0.88	0.66	1.17	.375	1.13	0.82	1.56	.444
All sexually transmitted disease/school/teen clinics or other facilities	0.74	0.42	1.31	.304	1.02	0.55	1.90	.949
Mixed type	0.94	0.65	1.34	.722	1.27	0.89	1.83	.19
All public facilities	(Referent)	N/A	N/A	N/A	(Referent)	N/A	N/A	N/A
Health-care professional orders HPV vaccine from the state								
Some but possibly or definitely not all healthcare professionals	1.07	0.75	1.52	.724	0.99	0.73	1.33	.929
No healthcare professionals	0.68	0.53	0.87	.002	0.76	0.60	0.95	.015
All healthcare professionals	(Referent)	N/A	N/A	N/A	(Referent)	N/A	N/A	N/A
**Macro-level characteristic**								
State religious ideology								
High religiosity	0.63	0.50	0.78	<.001	0.69	0.56	0.85	<.001
Moderate religiosity	0.68	0.55	0.85	.001	0.74	0.61	0.91	.004
Low religiosity	(Referent)	N/A	N/A	N/A	(Referent)	N/A	N/A	N/A

^a^AOR = adjusted odds ratio; CI = confidence interval; HPV = human papillomavirus; N//A = not applicable.

At the meso level, when none of the teen’s health-care professionals ordered vaccine from the state, teens had 32% lower odds of initiation (AOR = 0.68, 95% CI = 0.53 to 0.87, *P* = .002) than when all the teen’s health-care professionals ordered vaccine from the state.

At the individual level (Table 2), we found that teens identified as non-Hispanic Black or Hispanic, living in a household with a family income below the federal poverty level, who had had a well-child exam at 11-12 years of age, and those receiving a doctor’s recommendation for the HPV vaccine had higher odds of HPV vaccination initiation. Association between age and increased odds of initiation was also statistically significant. Teens whose mothers had 12 years of more of education (noncollege graduates and college graduates), however, had lower odds of HPV vaccine initiation than teens whose mothers had less than 12 years of education. Finally, sex and facility type were not statistically significant factors for HPV vaccine initiation.

### Up-to-date

Overall, the rate of being up-to-date with the HPV vaccination series among adolescents was 58.6% (Table 1). The up-to-date prevalence among teens was lowest in states with high religiosity (53.5%), and the prevalence of up-to-date was highest when all healthcare professionals ordered vaccine from the state or local health department (60.9%) (Table 1; Supplementary Figure 2, available online).

In the multivariable regression (Table 2), at the macro level, we found that teens living in states with high religiosity had 31% lower odds (AOR = 0.69, 95% CI = 0.56 to 0.85, *P* < .001) and moderate religiosity had 26% lower odds of being up-to-date (AOR = 0.74, 95% CI = 0.61 to 0.91, *P* = .004) than teens living in states will low religiosity. At the meso level, teens had 24% lower odds of being up-to-date (AOR = 0.76, 95% CI = 0.60 to 0.95, *P* = .015) when none of the teen’s health-care professionals ordered vaccine from the state or local health department than when all health-care professionals of the teen ordered vaccine from the state or local health department. At the individual level, teens who were non-Hispanic Black or Hispanic, lived in households below the federal poverty level, had received a well-child examination at 11-12 years of age, and had received a doctor’s recommendation for the HPV vaccine had higher odds of being up-to-date. The association between age and being up-to-date with HPV vaccination was also found statistically significant. Finally, sex, mother’s education level, and facility type were not statistically significant variables for being up-to-date with HPV vaccination (Table 2).

## Discussion

Using the nationally representative data from NIS-Teen 2020, we found in our study that approximately 25% of the US adolescent population between 13 and 17 years of age had yet to receive a single dose of the HPV vaccine, and only about 59% had completed the dose series. To investigate the factors associated with low initiation of and being up-to-date with HPV vaccination, the study was grounded in the Social Ecological theory, and we found that not only individual-level factors, such as age, race, ethnicity, family income, mother’s education level, having had a well child exam, and physician recommendation affected adolescent HPV vaccination coverage, but the macro-level and meso-level forces, such as state religiosity and source of vaccine procurement, played a significant role in adolescent HPV vaccine coverage.

At the macro level, we found that religiosity was a significant determinant of HPV vaccination coverage, after adjusting for other individual and organization-level characteristics. These findings are consistent with a study based on 2016 NIS-Teen data (35). Furthermore, the findings resonate with the existing literature at the individual level, such as highly religious parents having less vaccine intention (17) and high immunization refusal (36).

Individuals tend to be influenced by subjective norms based on the strength of the collective self or consciousness (37). Religion shapes collective consciousness, which means that followers not only have shared beliefs, attitudes, and norms that they integrate into their lives, but as a group, they also share resources and knowledge (38). Therefore, individuals living in states with conservative religious ideology feel hesitant to use HPV vaccination because, in the collective consciousness, the vaccine is perceived as an enabler of sexual promiscuity (as HPV infection is sexually transmitted) and perceived as containing religiously prohibited materials or processed in religiously unapproved ways (14,39). In contrast, other nonmandatory vaccines, such as SARS-CoV-2 and influenza, were not linked to religious beliefs associated with sexual norms and taboos. Among conservative religious groups, hesitancy regarding SARS-CoV-2 and influenza vaccine uptake was associated with mistrust of science, skepticism about vaccine effectiveness, and fear of serious side effects (40,41).

The religious reasons for declining vaccination were not driven by theology but by concerns about vaccine safety and religious beliefs among the social network of people in the faith community (42). Moreover, Williams and colleagues (41) found that most members of the clergy did not identify any vaccine prohibiting religious beliefs but “emphasized the religious beliefs highlighting preservation of life and community” through vaccination. Such findings indicate that religious leaders can become effective advocates for HPV vaccination by addressing the gap between theology and psychosocial barriers, particularly in religiously conservative states (43). State-medical-religious partnerships with faith-based organizations were proven effective in addressing community mistrust and facilitating the distribution of vaccines during COVID-19 (44). Faith-based interventions have been shown to be effective in improving community health behavior, such as hepatitis B virus testing and vaccination, intent to undergo yearly mammograms, and reducing HIV stigma and mistrust (45-47). Therefore, the findings indicate an opportunity for faith-based intervention in highly religious states to increase HPV vaccine coverage by increasing awareness and reducing misinformation.

Among the meso-level factors, we found a low likelihood of HPV vaccine coverage for teens when their clinicians did not order vaccine from the state (ie, not enrolled in the VFC program). This finding corroborates studies indicating that as more physicians enrolled in the VFC program, their cost-related barriers were reduced significantly, encouraging them to recommend the HPV vaccine (29). We also found that about a quarter of the healthcare professionals did not enroll in the VFC program. Expanding healthcare professional participation in the VFC program will increase HPV vaccine recommendation and coverage. Nonparticipation in the VFC program among healthcare professionals can be reduced by implementing policies that enable adequate and timely reimbursement of vaccine administration costs, covering costs associated with vaccine storage and recordkeeping, and educating healthcare professionals on the benefits of the VFC program (29,48-50). Our study found no significant association between facility type and vaccine coverage.

After controlling for the macro-level factor (state religiosity) and meso-level factors (VFC program enrollment and facility types), we found that at the individual level, the healthcare professional’s recommendation had the greatest impact on increasing the odds of initiation as well as being up-to-date compared with all the factors studied in the model. This finding resonates with the existing literature, which found that healthcare professional recommendation was statisticaly significant and positively associated with HPV vaccine initiation and being up-to-date (11,25,34,51). Lack of healthcare professional recommendation can be attributed to many factors, such as vaccine finance (ie, vaccine cost, inadequate reimbursement, and burden of determining insurance coverage) and the lack of training on the HPV vaccine (20,52). Therefore, the study findings indicated the need for interventions to improve physician knowledge and reduce the barriers to reimbursement for vaccine costs. We also found that at the individual level, teens were more likely to receive the HPV vaccination series if they were non-Hispanic Black or Hispanic, had a family income below the federal poverty level, and had had the well-child examination at 11-12 years of age. These findings were consistent with the current literature (8,53). We also found that teens with mothers who had 12 or more years of education were less likely to initiate the vaccination series. The inverse relationship between HPV vaccine uptake and household income and maternal education in our study corroborates the existing literature. Although the ability to prevent cervical cancer motivates low-income parents to use the HPV vaccine (54), concerns about vaccine safety among mothers with a high level of education were the main factor for HPV vaccine refusal (13). This finding calls for tailored interventions for mothers because they are the primary caregiver of most adolescent minor children (55,56).

This study had some limitations. Because it was a cross-sectional study, causality cannot be determined. Moreover, each teen’s household data were based on self-report, which increased the risk of social desirability and recall bias. Furthermore, religiosity was at the state level because NIS-Teen 2020 did not collect data on religiosity. The significant inverse association between state-level religiosity and individual-level HPV vaccine uptake found in our study, however, reaffirmed that religiosity shapes the collective consciousness of society at large, which in turn affects individual vaccine decisions. The response rate of NIS-Teens 2020 national data was low, but the NIS-Teen 2020 study incorporated weights adjusting for nonresponse for household interviews and insufficient clinician data (57). Importantly, the findings are nationally representative and can be generalized to the US population. In addition, the data on religiosity did not specify the types of religious affiliation (Christianity, Judaism, Islam, etc) or denominations within a religion (Protestant, Catholic, Lutheran, etc) in a state. Therefore, in our study, HPV vaccination coverage was not adjusted for heterogeneity in religious affiliations and intrareligion denominational variation across the states, which will be pertinent for future studies.

HPV vaccination can reduce the incidence of HPV-associated cancers if the population receives the recommended dosage. Rising oropharyngeal cancer rates and existing challenges with high mortality from cervical cancer call for more consolidated efforts to increase HPV vaccination initiation and being up-to-date. Based on our findings, we recommend a multiprong approach that includes a faith-based HPV vaccine intervention involving religious leaders, reduces the administrative barriers in vaccine cost reimbursement, and provides training to the healthcare community to increase vaccine recommendation.

## Supplementary Material

pkad068_Supplementary_Data

## Data Availability

The NIS-Teen 2020 data used in this manuscript are publicly available, and can be obtained from the NIS-Teen website (https://www.cdc.gov/vaccines/imz-managers/nis/datasets-teen.html).

## References

[1] Serrano B , Brotons M, Bosch FX, et al Epidemiology and burden of HPV-related disease. Best Pract Res Clin Obstet Gynaecol. 2018;47:14-26. 29037457 10.1016/j.bpobgyn.2017.08.006

[2] Centers for Disease Control and Prevention. *Cancers Associated with Human Papillomavirus, United States—2015–2019 USCS Data Brief, no.31*. 2022a. https://www.cdc.gov/cancer/uscs/about/data-briefs/no31-hpv-assoc-cancers-UnitedStates-2015-2019.htm. Accessed March 16, 2023.

[3] Centers for Disease Control and Prevention. *Cancers Associated with Human Papillomavirus, United States—2015–2019. USCS Data Brief, no.32*. 2022b. https://www.cdc.gov/cancer/uscs/about/data-briefs/no31-hpv-assoc-cancers-UnitedStates-2015-2019.htm. Accessed March 16, 2023.

[4] National Cancer Institute. *There are Vaccines that Can Prevent Cancer*. 2018. https://prevention.cancer.gov/news-and-events/infographics/there-are-vaccines-can-prevent-cancer-and-reduce-cancer-risk. Accessed April 24, 2023.

[5] Shimabukuro TT , Su JR, Marquez PL, et al Safety of the 9-valent human papillomavirus vaccine. Pediatrics (Evanston). 2019;144(6):e20191791. 10.1542/peds.2019-1791 PMC6935554 31740500

[6] Centers for Disease Control and Prevention. *Cancers Caused by HPV*. 2022c. https://www.cdc.gov/hpv/parents/cancer.html. Accessed February 23, 2023.

[7] Meites E , Kempe A, Markowitz LE. Use of a 2-dose schedule for human papillomavirus vaccination — updated recommendations of the Advisory Committee on Immunization Practices. MMWR Morb Mortal Wkly Rep. 2016;65(49):1405-1408. 27977643 10.15585/mmwr.mm6549a5

[8] Chido-Amajuoyi OG , Talluri R, Wonodi C, Shete S. Trends in HPV vaccination initiation and completion within ages 9-12 years: 2008-2018. Pediatrics. 2021;147(6):e2020012765. 10.1542/peds.2020-012765 PMC8785751 33941585

[9] Healthy People 2030. *Increase the Proportion of Adolescents Who Get Recommended Doses of the HPV Vaccine — IID-08*. https://health.gov/healthypeople/objectives-and-data/browse-objectives/vaccination/increase-proportion-adolescents-who-get-recommended-doses-hpv-vaccine-iid-08. Accessed April 1, 2023.

[10] Pingali C , Yankey D, Elam-Evans LD, et al National, regional, state, and selected local area vaccination coverage among adolescents aged 13–17 years—United States 2020. MMWR Morb Mortal Wkly Rep. 2021;70(35):1183-1190. 34473682 10.15585/mmwr.mm7035a1 PMC8422873

[11] Lu P-J , Yankey D, Fredua B, et al Human papillomavirus vaccination trends among adolescents: 2015 to 2020. Pediatrics. 2022;150(1):e2022056597. 10.1542/peds.2022-056597 PMC10961167 35730334

[12] Chido-Amajuoyi OG , et al The influence of parent-child gender on intentions to refuse HPV vaccination due to safety concerns/side effects, National Immunization Survey - Teen, 2010-2019. Hum Vaccin Immunother. 2022;18(5):2086762. 35797721 10.1080/21645515.2022.2086762 PMC9621054

[13] Chido-Amajuoyi OG , Talluri R, Shete SS, Shete S. Safety concerns or adverse effects as the main reason for human papillomavirus vaccine refusal: National Immunization Survey–Teen, 2008 to 2019. JAMA Pediatr. 2021;175(10):1074-1076. 34180965 10.1001/jamapediatrics.2021.1585 PMC8240004

[14] Bodson J , Wilson A, Warner EL, et al Religion and HPV vaccine-related awareness, knowledge, and receipt among insured women aged 18-26 in Utah. PLoS One. 2017;12(8):e0183725. 28841681 10.1371/journal.pone.0183725 PMC5571930

[15] Garcia LL , Yap JFC. The role of religiosity in COVID-19 vaccine hesitancy. J Public Health (Oxf). 2021;43(3):e529-e530. 34080617 10.1093/pubmed/fdab192 PMC8195070

[16] Wombwell E , Fangman MT, Yoder AK, et al Religious barriers to measles vaccination. J Community Health. 2015;40(3):597-604. 25315714 10.1007/s10900-014-9956-1

[17] Barnack JLP , Reddy DMP, Swain CMS. Predictors of parents' willingness to vaccinate for human papillomavirus and physicians' intentions to recommend the vaccine. Womens Health Issues. 2010;20(1):28-34. 20123174 10.1016/j.whi.2009.08.007

[18] Constantine NA , Jerman P. Acceptance of human papillomavirus vaccination among Californian parents of daughters: a representative statewide analysis. J Adolesc Health. 2007;40(2):108-115. 17259050 10.1016/j.jadohealth.2006.10.007

[19] Bronfenbrenner U. Ecological models of human development. In: *International Encyclopedia of Education*. Vol. 3. Oxford: Elsevier; 1994:37-43.

[20] Keating KM , Brewer NT, Gottlieb SL, et al Potential barriers to HPV vaccine provision among medical practices in an area with high rates of cervical cancer. J Adolesc Health. 2008;43(suppl 4):S61-S67. 18809147 10.1016/j.jadohealth.2008.06.015

[21] Williams CL , Walker TY, Elam-Evans LD, et al Factors associated with not receiving HPV vaccine among adolescents by metropolitan statistical area status, United States, National Immunization Survey–Teen, 2016–2017. Hum Vaccin Immunother. 2020;16(3):562-572. 31584312 10.1080/21645515.2019.1670036 PMC7227662

[22] Adjei Boakye E , Tobo BB, Rojek RP, et al Approaching a decade since HPV vaccine licensure: racial and gender disparities in knowledge and awareness of HPV and HPV vaccine. Hum Vaccin Immunother. 2017;13(11):2713-2722. 28853980 10.1080/21645515.2017.1363133 PMC5703403

[23] Sonawane K , Lin Y-Y, Damgacioglu H, et al Trends in human papillomavirus vaccine safety concerns and adverse event reporting in the United States. JAMA Netw Open. 2021;4(9):e2124502. 34533574 10.1001/jamanetworkopen.2021.24502 PMC8449282

[24] Amboree TL , Sonawane K, Deshmukh AA, et al Regular healthcare provider status does not moderate racial/ethnic differences in Human Papillomavirus (HPV) and HPV vaccine knowledge. Vaccines (Basel). 2021;9(7). 10.3390/vaccines9070802 PMC8310170 34358219

[25] Ejezie CL , Osaghae I, Ayieko S, et al Adherence to the recommended HPV vaccine dosing schedule among adolescents aged 13 to 17 years: findings from the National Immunization Survey-Teen, 2019–2020. Vaccines (Basel), 2022;10(4). doi:10.3390/vaccines10040577. PMC9026751 35455325

[26] National Conference of State Legislature. *States With Religious and Philosophical Exemptions From School Immunization Requirements*. 2022. https://www.ncsl.org/health/states-with-religious-and-philosophical-exemptions-from-school-immunization-requirements. Accessed April 3, 2023.

[27] Galbraith-Gyan KV , Lechuga J, Jenerette CM, et al HPV vaccine acceptance among African-American mothers and their daughters: an inquiry grounded in culture. Ethn Health, 2019;24(3):323-340. 28553758 10.1080/13557858.2017.1332758 PMC6175663

[28] Centers for Disease Control and Prevention. *Vaccine for Children Program (VFC)*. 2016. https://www.cdc.gov/vaccines/programs/vfc/about/index.html. Accessed April 2, 2023

[29] Lake PW , Kasting ML, Christy SM, et al Provider perspectives on multilevel barriers to HPV vaccination. Hum Vaccin Immunother. 2019;15(7-8):1784-1793. 30779687 10.1080/21645515.2019.1581554 PMC6746510

[30] Lu P-J , Yankey D, Jeyarajah J, et al HPV vaccination coverage of male adolescents in the United States. Pediatrics. 2015;136(5):839-849. 26504124 10.1542/peds.2015-1631 PMC5819004

[31] Centers for Disease Control and Prevention. *National Immunization Survey-Teen; A users Guide for the 2020 Pubic-Use Data File*. 2021. https://www.cdc.gov/vaccines/imz-managers/nis/downloads/NIS-TEEN-PUF20-DUG.pdf. Accessed June 13, 2022.

[32] Lipika M , Wormald B. *Religious Landscape Study*. 2014. https://www.pewresearch.org/fact-tank/2016/02/29/how-religious-is-your-state/?state=alabama. Accessed June 13, 2022.

[33] Elenwo C , Batioja K, Davis T, et al Associations of maternal age, education, and marital status with HPV vaccine uptake and hesitancy among United States youth: a cross-sectional analysis of the 2020 National Immunization Survey. J Pediatr Adolesc Gynecol. 2023;36(3):273-279. 36758721 10.1016/j.jpag.2023.01.213

[34] Osaghae I , Chido-Amajuoyi OG, Shete S. Healthcare provider recommendations and observed changes in HPV vaccination acceptance during the COVID-19 pandemic. Vaccines (Basel). 2022;10(9):1515. 10.3390/vaccines10091515 PMC9504052 36146593

[35] Franco M , Mazzucca S, Padek M, et al Going beyond the individual: how state-level characteristics relate to HPV vaccine rates in the United States. BMC Public Health. 2019;19(1):246. 30819149 10.1186/s12889-019-6566-y PMC6393974

[36] Shelton RC , Snavely AC, De Jesus M, et al HPV vaccine decision-making and acceptance: does religion play a role? J Relig Health. 2013;52(4):1120-1130. 22076049 10.1007/s10943-011-9553-x PMC4616263

[37] Ybarra O , Trafimow D. How priming the private self or collective self affects the relative weights of attitudes and subjective norms. Pers Soc Psychol Bull. 1998;24(4):362-370.

[38] Durkheim É , Pickering WSF. *Durkheim on Religion: A Selection of Readings with Bibliographies and Introductory Remarks*. United Kingdom: James Clarke & Company Limited; 2011:1-390.

[39] Hendry M , Lewis R, Clements A, Damery S, Wilkinson C. “HPV? Never heard of it!”: a systematic review of girls’ and parents’ information needs, views and preferences about human papillomavirus vaccination. Vaccine. 2013;31(45):5152-5167. 24029117 10.1016/j.vaccine.2013.08.091

[40] Troiano G , Nardi A. Vaccine hesitancy in the era of COVID-19. Public Health. 2021;194:245-251. 33965796 10.1016/j.puhe.2021.02.025 PMC7931735

[41] Williams JTB , Fisher MP, Bayliss EA, et al Clergy attitudes toward vaccines and vaccine advocacy: a qualitative study. Hum Vaccin Immunother. 2020;16(11):2800-2808. 32209009 10.1080/21645515.2020.1736451 PMC7734036

[42] Grabenstein JD. What the world's religions teach, applied to vaccines and immune globulins. Vaccine. 2013;31(16):2011-2023. 23499565 10.1016/j.vaccine.2013.02.026

[43] Viskupič F , Wiltse DL. The messenger matters: religious leaders and overcoming COVID-19 vaccine hesitancy. APSC. 2022;55(3):504-509.

[44] Monson K , Oluyinka M, Negro D, et al Congregational COVID-19 conversations: utilization of medical-religious partnerships during the SARS-CoV-2 pandemic. J Relig Health. 2021;60(4):2353-2361. 34032973 10.1007/s10943-021-01290-x PMC8144273

[45] Bastani R , Glenn BA, Maxwell AE, et al Cluster-randomized trial to increase hepatitis B testing among Koreans in Los Angeles. Cancer Epidemiol Biomarkers Prev. 2015;24(9):1341-1349. 26104909 10.1158/1055-9965.EPI-14-1396 PMC4560609

[46] Ka'opua LSI , Park SH, Ward ME, et al Testing the feasibility of a culturally tailored breast cancer screening intervention with Native Hawaiian women in rural churches. Health Soc Work. 2011;36(1):55-65. 21446609 10.1093/hsw/36.1.55 PMC3070905

[47] Derose KP , Griffin BA, Kanouse DE, et al Effects of a pilot church-based intervention to reduce HIV stigma and promote HIV testing among African Americans and Latinos. AIDS Behav. 2016;20(8):1692-1705. 27000144 10.1007/s10461-015-1280-y PMC4945375

[48] Campos-Outcalt D , Jeffcott-Pera M, Carter-Smith P, et al Vaccines provided by family physicians. Ann Fam Med. 2010;8(6):507-510. 21060120 10.1370/afm.1185 PMC2975685

[49] Malo TL , Hassani D, Staras SAS, et al Do Florida Medicaid providers’ barriers to HPV vaccination vary based on VFC program participation? Matern Child Health J. 2013;17(4):609-615. 22569945 10.1007/s10995-012-1036-5 PMC3795412

[50] O'Leary ST , et al Vaccine financing from the perspective of primary care physicians. Pediatrics (Evanston). 2014;133(3):367-374. 10.1542/peds.2013-2637 PMC4731026 24567011

[51] Dorell CG , Yankey D, Santibanez TA, et al Human papillomavirus vaccination series initiation and completion, 2008-2009. Pediatrics (Evanston). 2011;128(5):830-839. 10.1542/peds.2011-0950 22007006

[52] Osaghae I , Darkoh C, Chido-Amajuoyi OG, et al Association of provider HPV vaccination training with provider assessment of HPV vaccination status and recommendation of HPV vaccination. Hum Vaccin Immunother. 2022;18(6):2132755. 36265005 10.1080/21645515.2022.2132755 PMC9746413

[53] Jeudin P , Liveright E, Del Carmen MG, et al Race, ethnicity, and income factors impacting human papillomavirus vaccination rates. Clin Ther. 2014;36(1):24-37. 24417783 10.1016/j.clinthera.2013.11.001

[54] Perkins RB , Pierre-Joseph N, Marquez C, et al Why do low-income minority parents choose human papillomavirus vaccination for their daughters? J Pediatr. 2010;157(4):617-622. 20472250 10.1016/j.jpeds.2010.04.013 PMC3459226

[55] Kose D , Erkorkmaz U, Cinar N, et al Mothers' knowledge and attitudes about HPV vaccination to prevent cervical cancers. Asian Pac J Cancer Prev. 2014;15(17):7263-7266. 25227825 10.7314/apjcp.2014.15.17.7263

[56] Panozzo CA , Head KJ, Kornides ML, et al Tailored messages addressing human papillomavirus vaccination concerns improves behavioral intent among mothers: a randomized controlled trial. J Adolesc Health. 2020;67(2):253-261. 32199723 10.1016/j.jadohealth.2020.01.024

[57] Centers for Disease Control and Prevention. *National Immunization Survey-Teen: A Users guide for the 2020 Public Use Data File*. 2020. https://www.cdc.gov/vaccines/imz-managers/nis/downloads/NIS-TEEN-PUF20-DUG.pdf. Accessed May 13, 2023.

