# Supplementary material for: Barriers to human papillomavirus vaccine uptake: role of state religiosity and healthcare professionals’ participation in a state vaccine program
Source: JNCI Cancer Spectr. 2023 Sep 12;7(5):pkad068. doi: 10.1093/jncics/pkad068 (PMC10575682; doi:10.1093/jncics/pkad068)

Supplementary Figure 1: HPV vaccine initiation by state religiosity: National Immunization survey–Teen 2020

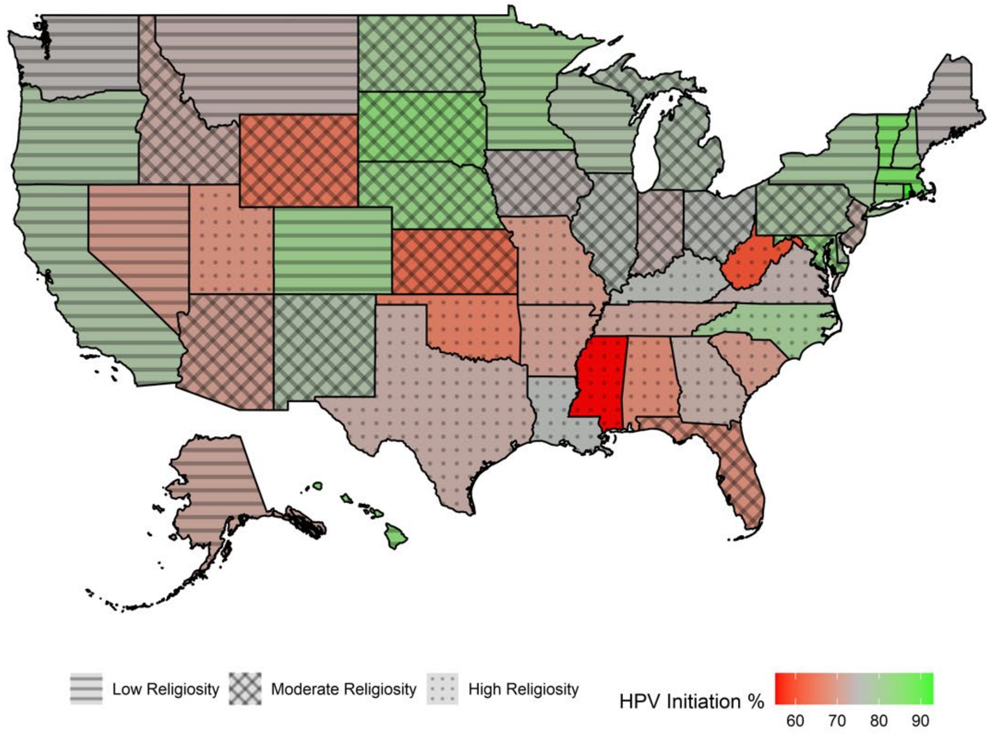

Supplementary Figure 2: HPV vaccine Up-To-Date (UTD) by state religiosity: National Immunization Survey–Teen 2020

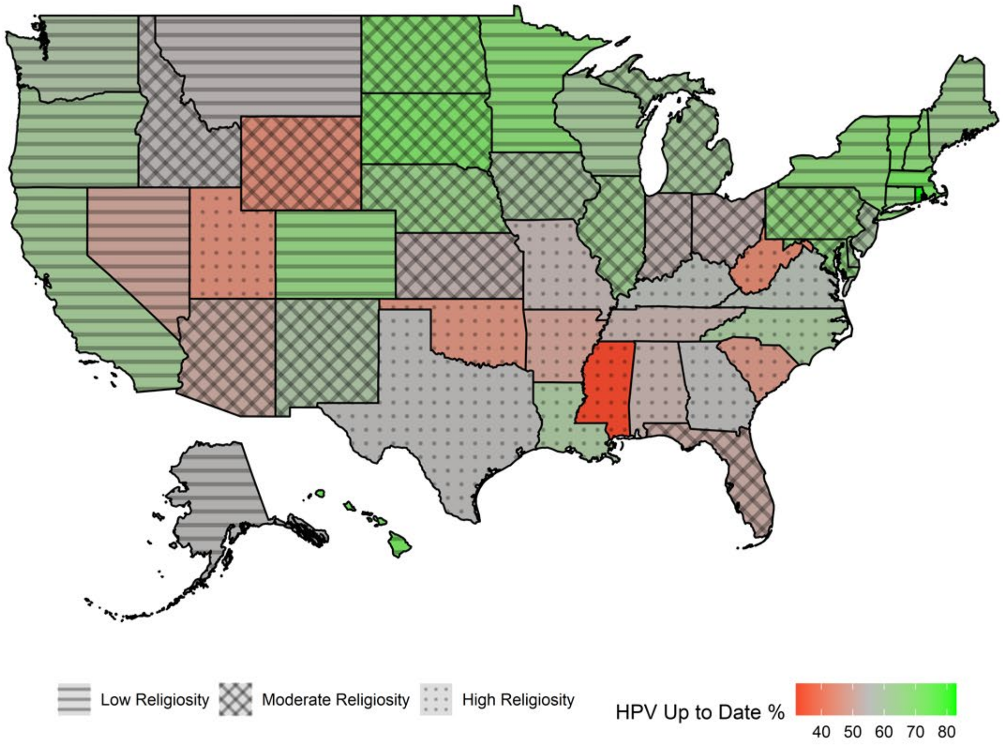

Supplement: pkad068_Supplementary_Data [file pkad068_supplementary_data.pdf]
